# The Impact of Glyphosate, Its Metabolites and Impurities on Viability, ATP Level and Morphological changes in Human Peripheral Blood Mononuclear Cells

**DOI:** 10.1371/journal.pone.0156946

**Published:** 2016-06-09

**Authors:** Marta Kwiatkowska, Paweł Jarosiewicz, Jaromir Michałowicz, Maria Koter-Michalak, Bogumiła Huras, Bożena Bukowska

**Affiliations:** 1 Department of Environmental Pollution Biophysics, University of Lodz, Lodz, Poland; 2 Institute of Industrial Organic Chemistry, Warsaw, Poland; Centro Cardiologico Monzino, ITALY

## Abstract

The toxicity of herbicides to animals and human is an issue of worldwide concern. The present study has been undertaken to assess toxic effect of widely used pesticide—glyphosate, its metabolites: aminomethylphosphonic acid (AMPA) and methylphosphonic acid and its impurities: *N*-(phosphonomethyl)iminodiacetic acid (PMIDA), *N*-methylglyphosate, hydroxymethylphosphonic acid and bis-(phosphonomethyl)amine on human peripheral blood mononuclear cells (PBMCs). We have evaluated the effect of those compounds on viability, ATP level, size (FSC-A parameter) and granulation (SSC-A parameter) of the cells studied. Human peripheral blood mononuclear cells were exposed to different concentrations of glyphosate, its metabolites and impurities (0.01–10 mM) for 4 and 24 h. It was found that investigated compounds caused statistically significant decrease in viability and ATP level of PBMCs. The strongest changes in cell viability and ATP level were observed after 24 h incubation of PBMCs with bis-(phosphonomethyl)amine, and particularly PMIDA. Moreover, all studied compounds changed cell granularity, while PMIDA and bis-(phosphonomethyl)amine altered PBMCs size. It may be concluded that bis-(phosphonomethyl)amine, and PMIDA caused a slightly stronger damage to PBMCs than did glyphosate. Changes in the parameters studied in PBMCs were observed only at high concentrations of the compounds examined, which clearly shows that they may occur in this cell type only as a result of acute poisoning of human organism with these substances.

## Introduction

Glyphosate (*N*-phosphonomethylglycine) is a total herbicide which destroys plants and microorganisms by inhibiting shikimate pathway, and thus it was considered as completely non-toxic to animals and human [1]. Glyphosate inhibits the activity of the enzyme synthase 5-enolpyruvylshikimate-3-phosphate synthase (EPSPS) by competitive inhibition, which prevents the production of chorismate that is a precursor for aromatic amino acids using in the synthesis of a number of pigments, flavonoids and anthocyanins [2,3].

According to the European Parliament and Council Regulation 1107/2009/EC on 21^st^ of October 2009, the studies conducted on human peripheral blood mononuclear cells (PBMCs) may be very important in the evaluation of toxic effects of glyphosate, its metabolites and impurities on the human body. PBMCs are components of the immune system and are used as cellular model. PBMCs participate in maintaining homeostasis in human body [4].

The analysis of adverse effects of metabolites and impurities of pesticides seems to be very important in evaluation of toxicological risk exerted by pesticide preparations. It has been proven that metabolites and impurities of the pesticides often reveal stronger toxicity than their parent compounds [5, 6]. Despite of the existing norms of application, glyphosate as well as and its metabolites or impurities enter the environment, contaminating water, soil and food and, thus pose a risk to human health.

**Fig 1:**
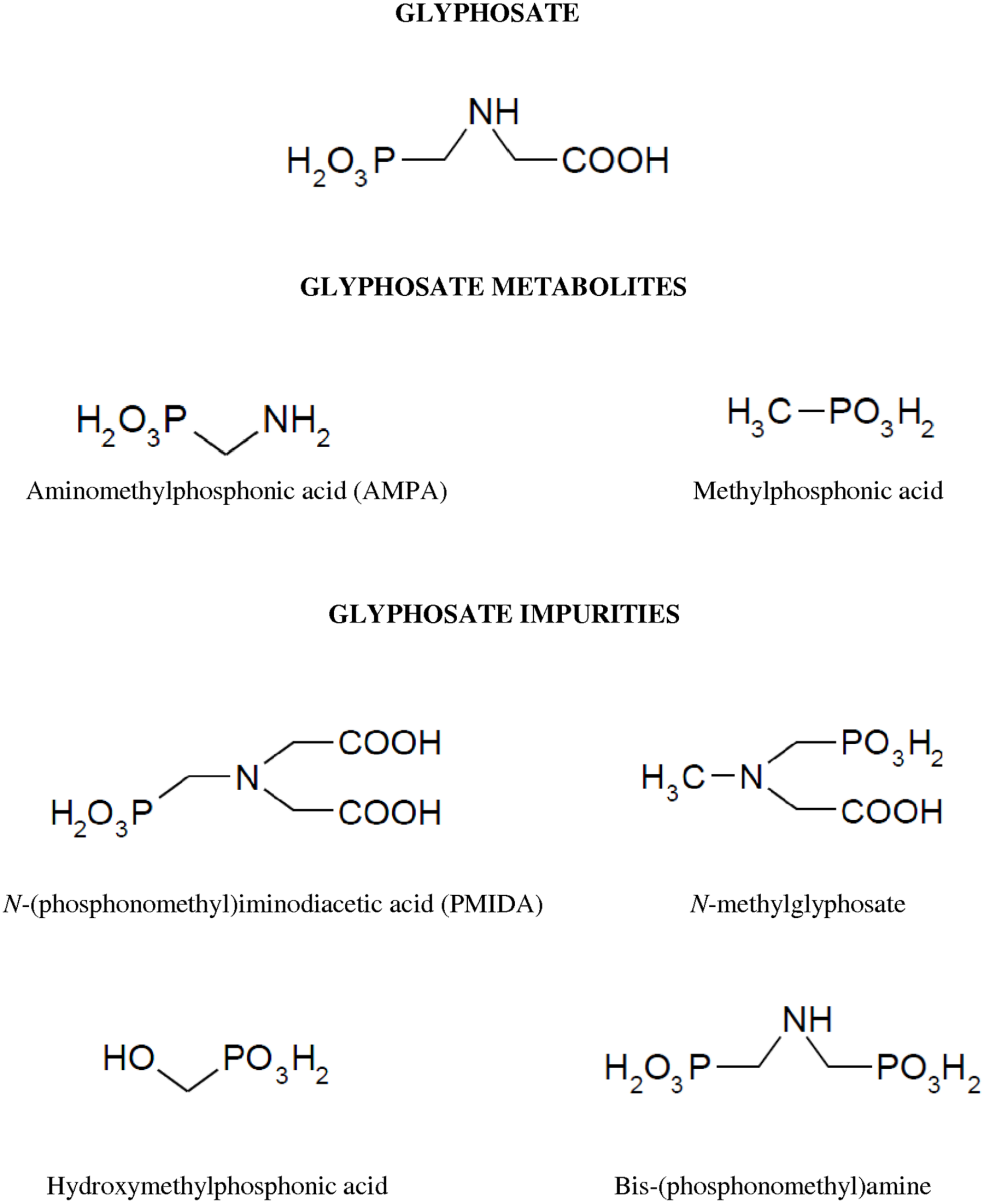
Chemical structure of glyphosate, its metabolites—aminomethylphosphonic acid (AMPA), methylphosphonic acid and impurities–*N*-(phosphonomethyl)iminodiacetic acid (PMIDA), *N*-methylglyphosate, hydroxymethylphosphonic acid and bis-(phosphonomethyl)amine.

In this study, we have assessed the effect of glyphosate and its metabolites: aminomethylphosphonic acid (AMPA) and methylphosphonic acid on PBMCs. AMPA is a primary degradation product of glyphosate that is formed under the action of microorganisms [7] and as a result of oxidative cleavage of glyphosate by glyphosate oxidoreductase (GOX) [8]. Plants, which are resistant to glyphosate, metabolize it to AMPA [9], which exhibits much higher mobility in the soil than a glyphosate [10]. Glyphosate preparations can also contain many of types of impurities. One of them is *N*-(phosphonomethyl)iminodiacetic acid (PMIDA), which is a substrate used in the production of glyphosate [11]. The second examined by-product of glyphosate *N*-methylglyphosate is formed during oxidation of PMIDA [12]. In this study, hydroxymethylphosphonic acid and bis-(phosphonomethyl)amine have also been examined as potential impurities of glyphosate [13]. We have analyzed the effect of low concentrations (0.01 mM) of the xenobiotics studied on human PBMCs (environmental exposure), as well as high concentrations of these substances, which may enter human organism only as a result of acute poisoning (> 0.05 mM) (Fig 1).

## Materials and Methods

### Chemicals

The investigated compounds i.e., aminomethylphosphonic acid (AMPA) (purity 98%), methylphosphonic acid (purity 98%), *N*-(phosphonomethyl)iminodiacetic acid (PMIDA) (purity 98%), *N*-methylglyphosate, hydroxymethylphosphonic acid (purity 98%) and bis-(phosphonomethyl)amine (purity 97%) were provided by the Institute of Industrial Organic Chemistry, Warsaw, Poland. Glyphosate [*N*-(phosphonomethyl)glicine] (purity 95%) was bought from Sigma-Aldrich, USA. The investigated compounds were dissolved in phosphate-buffered saline (pH = 7.4). Other chemicals were purchased from POCh (Poland) and were of analytical grade.

### Human peripheral blood mononuclear cells isolation

PBMCs were isolated from leucocyte-buffy coat collected from blood obtained in Blood Bank in Lodz, Poland. Blood was obtained from healthy, non-smoking volunteers, who showed no signs of infection disease symptoms at the time the blood samples were collected. The testing was approved by the Bioethics Committee of the University of Lodz No. KBBN-UŁ/I/3/2013. PBMCs were diluted with PBS (1:4) and isolated using LSM (1.077 g/cm^3^) by centrifugation at 600 *g* for 30 min at 20°C. PBMCs were collected, suspended in erythrocyte lysis buffer (150 mM NH_4_Cl, 10 mM NaHCO_3_, 1 mM EDTA, pH 7.4) and incubated for 5 min at 20°C. Then, PBS was added immediately, and the cells were centrifuged at 200 *g* for 15 min at 20°C. The supernatant was decanted, and the cells were washed twice with RPMI with L-glutamine and 10% fetal bovine serum (FBS) at 200 *g* for 15 min. The cells were resuspended in RPMI medium with L-glutamine, 10% FBS and penicylin-streptomycin (0.5%) and counted in haemocytometer. The final PBMCs density used in the experiments (after addition of glyphosate, its metabolites or impurities) was 2x10^6^ cells/ml. The viability of the cells was over 95%.

### Cell viability (necrotic changes), calcein-AM/ propidum iodide staining

The calcein-AM/propidum iodide (PI) viability test is commonly used in toxicity assays to detect cell membrane integrity and to quantify the number of viable and necrotic cells. The calcein-AM is hydrolyzed to calcein, which has a negative charge and penetrates living cells, staining them green [14]. PI is one of the most widely used fluorescent markers for staining of necrotic cells. This compound penetrates the cells, in which membrane was damaged, and binds to DNA. This dye has two positive charges, which prevent it from entering intact cells [15]. PBMCs were incubated with glyphosate, its metabolites or impurities in the final concentrations ranging from 0.01 to 10 mM for 4 h and 24 h at 37°C in total darkness. After the incubation, the samples were centrifuged at 300 g for 5 min at 4°C, the supernatant was decanted, and the cells were supplemented with RPMI with L-glutamine and 10% FBS. The samples were treated with calcein-AM and PI in the final concentrations of 0.1 and 1 μM, respectively and incubated for 15 min at 37°C in total darkness. The analysis was performed by flow cytometry (Becton Dickinson, LSR II). FCM gate on PBMCs has been established for data acquisition, and the fluorescence was measured with excitation/emission maxima of 494/517 nm and 535/617 nm for calcein and PI, respectively. The data were recorded for a total of 10,000 events per sample.

### Determination of the level of adenosine-5'-triphosphate (ATP)

The level of ATP in the cells was determined by bioluminescence measurement at 560 nm using recombinant luciferase and its substrate, D-luciferin. The high sensitivity of method enables detection of 0.1 pM ATP. The reaction proceeds in two stages. In the first step, as a result of the reaction of ATP with luciferin, a complex of adenosine with luciferin is formed with simultaneous releasing of phosphate group. Secondly the complex reacts with oxygen resulting in a formation of oxyluciferin and adenosine monophosphate (AMP). Oxyluciferin returning from its excited state to the base state emits luminescence. The reaction requires the presence of magnesium ions.

### Measurement of cell morphology

Control cells and the cells treated with glyphosate, its metabolites or impurities were incubated for 4 h and 24 h at 37°C in total darkness. The cells were then analyzed using a flow cytometry (LSR II; Becton-Dickinson). FMC gate on PBMCs has been established for data acquisition, and cell size and granularity were evaluated with simultaneous separate detection of low-angle (FSC-A) and right-angle (SSC-A) light scattering. The data obtained were displayed in a form of a diagram of cell number versus light scatter and were analyzed using the standard computer program WinMDI2.8. The light scattered near the forward direction (low angle) is expected to be proportional to particle size (volume), whereas scattering at the right angle depends on cell granularity (internal properties of the scattered particles).

### Statistical analysis

The statistical analysis was performed with STATISTICA 8 data analysis software (2000 StatSoft, Inc., Tulsa, OK, USA). In this study, one-way analysis of variance (ANOVA) with post hoc multiple comparisons procedure (Tukey test) was used to assess statistical differences in case of normal distribution. The difference was considered to be significant for P<0.05. The individual analysis was performed on blood from 4 to 5 donors while each experiment was repeated trice.

## Results

### Cell viability (necrotic changes), calcein-AM/ propidum iodide staining

**Fig 2:**
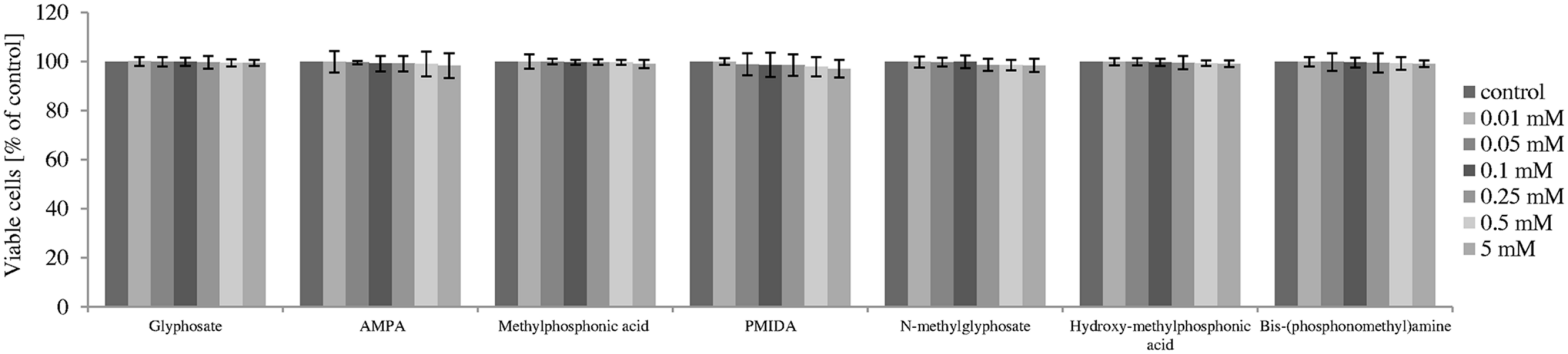
Changes in viability of human PBMCs incubated with glyphosate, its metabolites and impurities in the concentrations ranging from 0.5 to 10 mM for 4 h. The control sample was referred as 100%. (*) Significantly different from control (P < 0.05); one-way ANOVA and a posteriori Tukey test.

After 4 h incubation, a decrease in cell viability was not observed for any compound studied (Fig 2).

**Fig 3:**
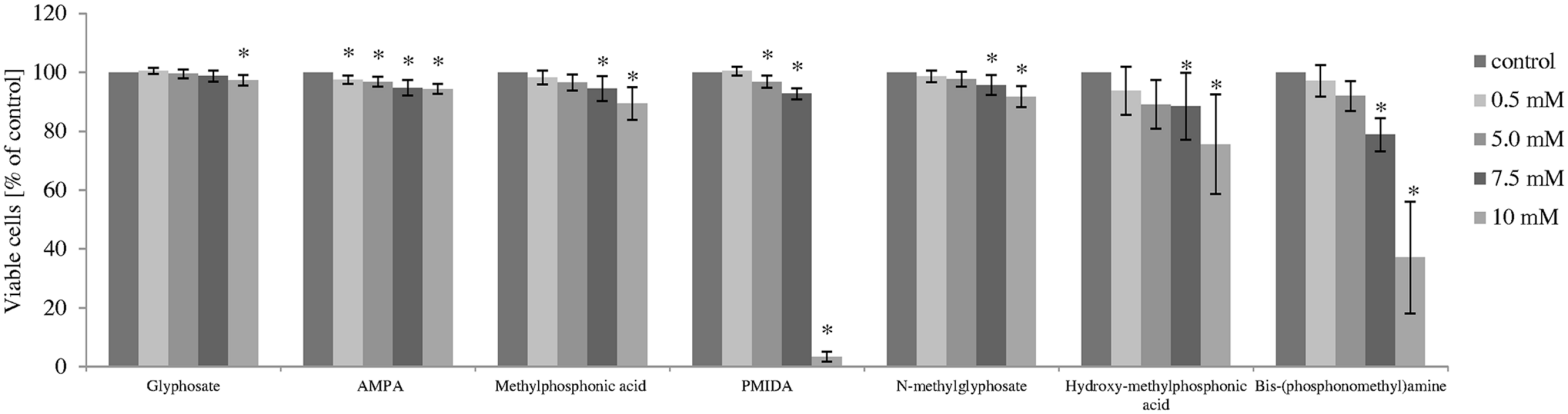
Changes in viability of human PBMCs incubated with glyphosate, its metabolites and impurities in the concentrations ranging from 0.5 to 10 mM for 24 h. The control sample was referred as 100%. (*) Significantly different from control (P < 0.05); one-way ANOVA and a posteriori Tukey test.

Statistically significant decrease in PBMCs viability was observed for all compounds studied after 24 h incubation (Fig 3).

Glyphosate showed very low cytoxicity because even at 10 mM it decreased cell viability only by 2.7% (F_4;55_ = 9.06; P < 0.001). The strongest adverse effects were noted for PMIDA from 5 mM (F_4;55_ = 8605.41; P < 0.001) and bis-(phosphonomethyl)amine from 7.5 mM (F_4;55_ = 89.62; P < 0.001). IC_50_ values estimated for these compounds were 7.9 mM for PMIDA and 8.7 mM for bis-(phosphonomethyl)amine (Table 1). Those substances at 10 mM decreased PBMCs viability by up to 3.4% and 37.2%, respectively. Hydroxymethylphosphonic acid, methylphosphonic acid and *N*-methylglyphosate caused a decrease in cell viability only at 7.5 mM and 10 mM (F_4;55_ = 7.45; P < 0.001; F_4;55_ = 16.24; P < 0.001; F_4;55_ = 17.48; P < 0.001), while AMPA decreased cell viability from 0.5 mM (F_4;55_ = 6.22; P < 0.001).

**Table 1:** IC_50_ values for changes in viability and ATP level in human PMBCs incubated with glyphosate, its metabolites and impurities for 24 h.

	IC 50	
	Viability	ATP level
Glyphosate	>10	9.6
AMPA	>10	>10
Methylphosphonic acid	>10	>10
PMIDA	7.9	6.1
*N*-methylglyphosate	>10	>10
Hydroxy-methylphosphonic acid	>10	>10
Bis-(phosphonomethyl)amine	8.7	5.3

### Determination of the level of adenosine-5'-triphosphate (ATP)

**Fig 4:**
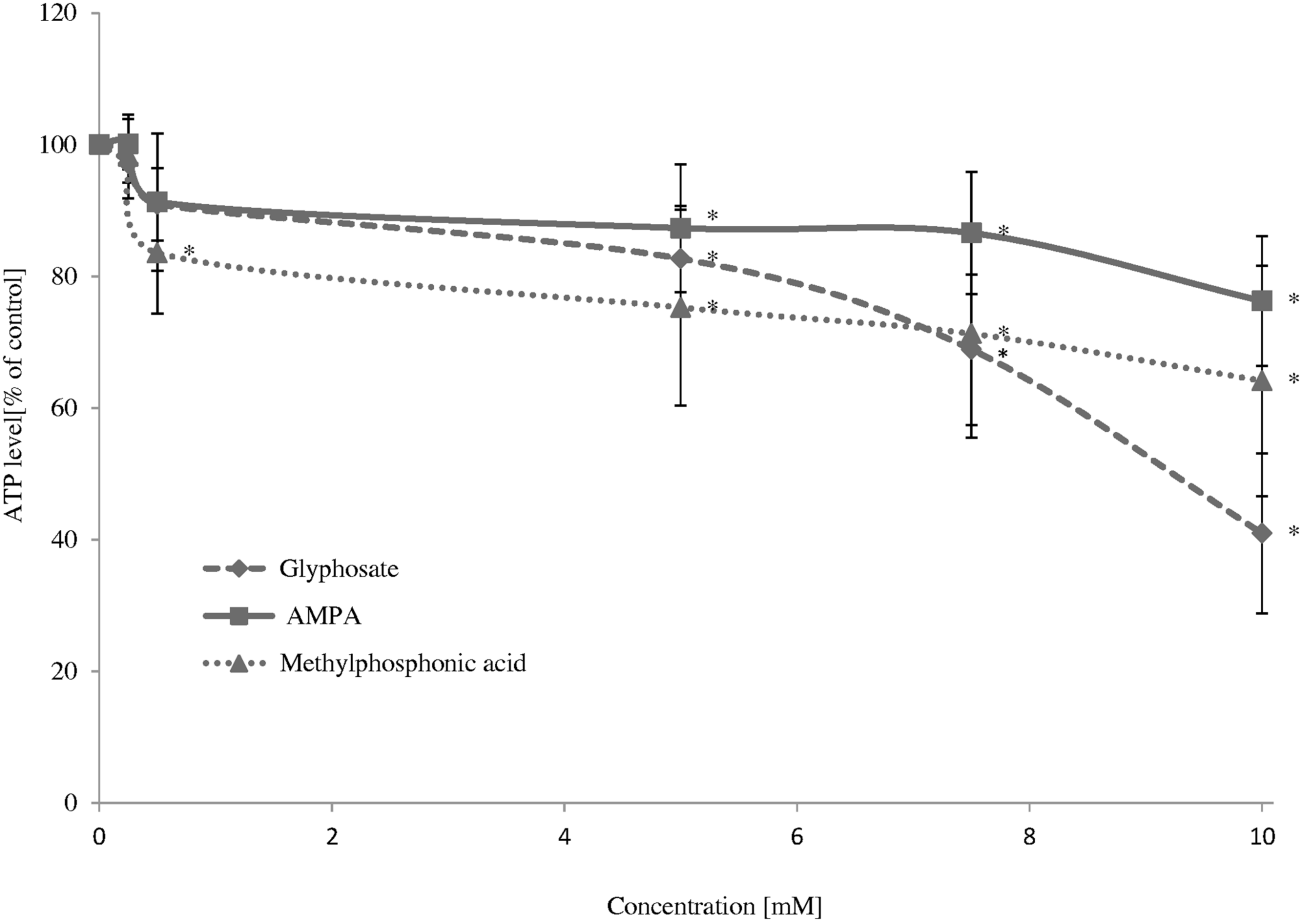
Changes in ATP level in control human PBMCs and cells incubated with glyphosate and its metabolites in the concentrations ranging from 0.25 to 10 mM for 24 h. The control sample was referred as 100%. (*) Significantly different from control (P < 0.05); one-way ANOVA and a posteriori Tukey test.

**Fig 5:**
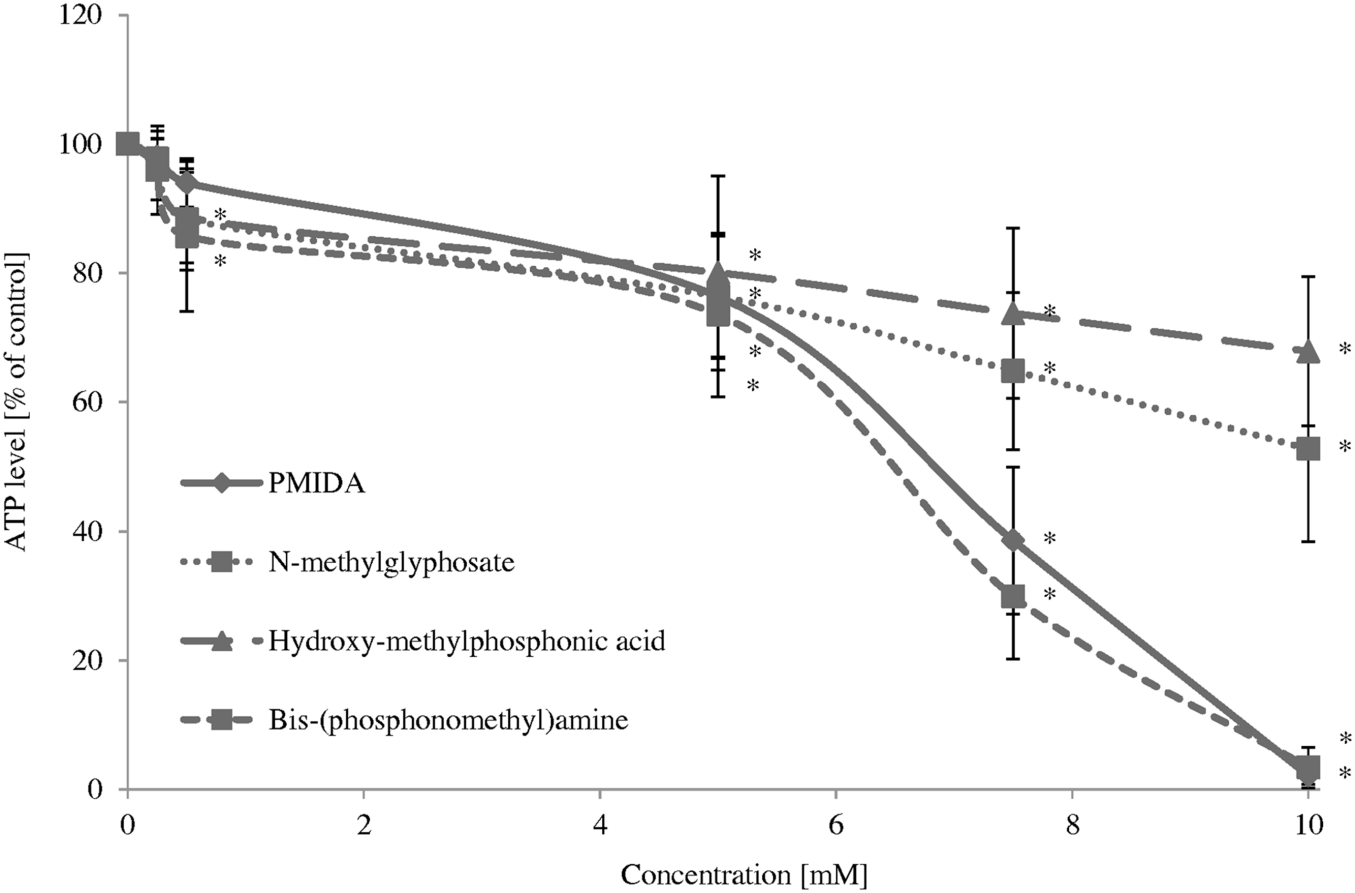
Changes in ATP level in control human PBMCs and cells incubated with glyphosate and its impurities in the concentrations ranging from 0.25 to 10 mM for 24 h. The control sample was referred as 100%. (*) Significantly different from control (P < 0.05); one-way ANOVA and a posteriori Tukey test.

A decrease in ATP level was observed in cells treated with glyphosate from 5 mM (F_5;66_ = 93.27; P < 0.001), AMPA (F_5;66_ = 14.82; P < 0.001), PMIDA (F_5;66_ = 461.50; P < 0.001) and hydroxymethylphosphonic acid (F_5;66_ = 18.87; P < 0.001) and for methylphosphonic acid (F_5;66_ = 15.64; P < 0.001), *N*-methylglyphosate (F_5;66_ = 49.55; P < 0.001) and bis-(phosphonomethyl)amine (F_5;66_ = 270.06; P < 0.001) from 0.5 mM after 24 h. The strongest effects were observed for PMIDA and bis-(phosphonomethyl)amine at 10 mM. Those compounds at 10 mM decreased ATP level by up to 2.1% and 3.4%, respectively (Figs 4 and 5).

### Measurement of cell morphology

**Fig 6:**
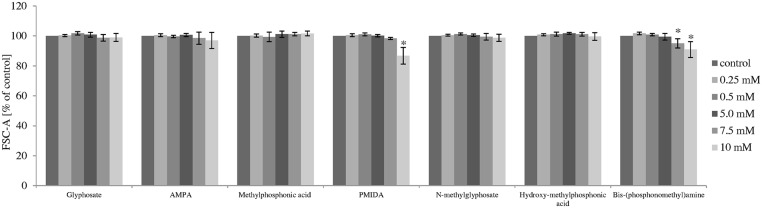
Flow cytometry analysis of changes in the size (FSC-A parameter) of control human PBMCs and cells incubated with glyphosate, its metabolites and impurities in the concentrations ranging from 0.25 to 10 mM for 24 h. (*) Significantly different from control (P < 0.05); one-way ANOVA and a posteriori Tukey test.

**Fig 7:**
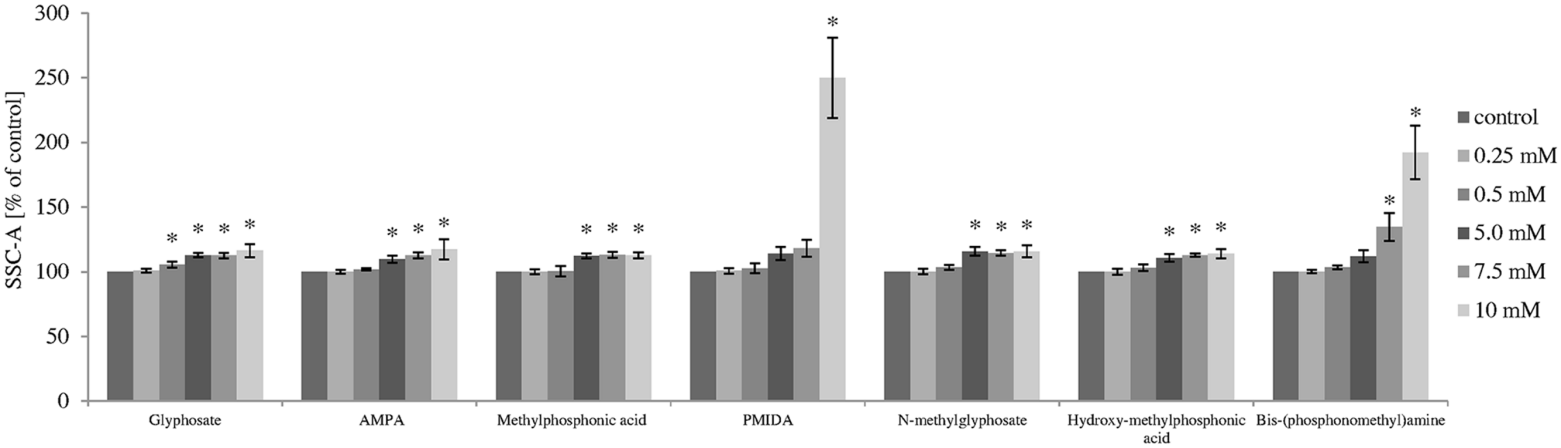
Flow cytometry analysis of changes in the granularity (SSC-A parameter) of control human PBMCs and cells incubated with glyphosate, its metabolites and impurities in the concentrations ranging from 0.25 to 10 mM for 24 h. (*) Significantly different from control (P < 0.05); one-way ANOVA and a posteriori Tukey test.

Flow cytometry technique was used to analyze the size and the granularity of PBMCs. Table 2 and Figs 6 and 7 represent quantitative changes in FSC-A and SSC-A parameters after 4 and 24 h incubation.

**Table 2:** Changes (expressed in percent) in FSC-A and SSC-A parameters of control human PBMCs and cells incubated with glyphosate, its metabolites and impurities in the concentrations ranging from 0.01 to 5 mM for 4 h.

Compounds	Concentration (mM)	FSC-A	SSC-A
Glyphosate	control	100	100
	0.01	102.0 ± 3.55	99.2 ± 4.71
	0.05	101.6 ± 2.94	99.7 ± 7.75
	0.1	101.8 ± 2.38	100.6 ± 9.59
	0.25	101.8 ± 4.69	100.6 ± 8.10
	0.5	101.9 ± 3.89	101.6 ± 5.94
	5.0	100.7 ± 3.00	104.3 ± 7.27
	ANOVA	*F*_6;119_ = 1.00; *P*>0.05	*F*_6;119_ = 0.35; *P*>0.05
Aminomethyl-phosphonic acid (AMPA)	control	100	100
	0.01	101.2 ± 4.61	101.1 ± 3.75
	0.05	101.3 ± 4.45	100.2 ± 3.47
	0.1	101.0 ± 5.04	100.6 ± 2.26
	0.25	100.8 ± 3.96	101.1 ± 5.74
	0.5	101.8 ± 3.93	101.3 ± 5.35
	5.0	99.6 ± 5.18	102.4 ± 4.76
	ANOVA	*F*_6;112_ = 0.54; *P*>0.05	*F*_6;112_ = 0.76; *P*>0.05
Methylphosphonic acid	control	100	100
	0.01	100.0 ± 2.97	98.5 ± 3.85
	0.05	101.3 ± 3.19	99.6 ± 1.17
	0.1	100.7 ± 2.84	99.9 ± 1.42
	0.25	102.5 ± 2.57	99.9 ± 1.24
	0.5	101.7 ± 2.98	101.9 ± 3.21
	5.0	100.9 ± 2.44	104.1 ± 2.3*
	ANOVA	*F*_6;77_ = 1.42; *P*>0.05	*F*_6;77_ = 8.14; *P*<0.001
*N*-(phosphonomethyl) iminodiacetic acid (PMIDA)	control	100	100
	0.01	100.4 ± 2.09	97.5 ± 4.97
	0.05	100.0 ± 2.21	98.1 ± 5.81
	0.1	100.3 ± 3.36	99.2 ± 5.42
	0.25	100.1 ± 2.65	99.3 ± 4.63
	0.5	100.5 ± 2.65	99.8 ± 6.30
	5.0	100.6 ± 4.59	104.6 ± 5.12
	ANOVA	*F*_6;119_ = 0.16; *P*>0.05	*F*_6;119_ = 3.81; *P*>0.05
*N*-methylglyphosate	control	100	100
	0.01	101.5 ± 3.97	101.0 ± 4.68
	0.05	102.1 ± 2.66	101.3 ± 4.81
	0.1	102.6 ± 3.27	101.5 ± 3.57
	0.25	100.7 ± 5.72	101.9 ± 4.73
	0.5	100.8 ± 4.66	101.8 ± 8.82
	5.0	100.5 ± 4.82	102.1 ± 7.27
	ANOVA	*F*_6;112_ = 0.93; *P*>0.05	*F*_6;112_ = 0.30; *P*>0.05
Hydroxymethylo-phosphonic acid	control	100	100
	0.01	100.4 ± 2.00	96.8 ± 4.77
	0.05	100.2 ± 1.84	97.6 ± 4.71
	0.1	100.1 ± 1.91	99.4 ± 1.17
	0.25	100.7 ± 3.68	100.5 ± 3.33
	0.5	100.2 ± 2.40	100.6 ± 2.05
	5.0	100.5 ± 2.94	101.7 ± 2.14
	ANOVA	*F*_6;77_ = 0.14; *P*>0.05	*F*_6;77_ = 3.91; *P*>0.05
Bis-(phophonomethyl)-amine	control	100	100
	0.01	100.6 ± 2.38	98.4 ± 2.94
	0.05	99.7 ± 2.54	100.0 ± 2.55
	0.1	99.6 ± 2.04	100.1 ± 3.68
	0.25	100.1 ± 2.68	100.5 ± 2.00
	0.5	100.7 ± 3.34	101.1 ± 1.12
	5.0	100.3 ± 3.88	102.8 ± 3.17
	ANOVA	*F*_6;77_ = 0.31; *P*>0.05	*F*_6;77_ = 3.33; *P*>0.05

^(^*^)^ Significantly different from control (P < 0.05); one-way ANOVA I and a posteriori Tukey test.

The analysis of FSC-A parameter allowed obtaining information about PBMCs size, while the SSC-A parameter provides the information about cell granularity.

After 4 h incubation, glyphosate, its metabolites and its impurities did not change FSC-A parameter, while changes in SSC-A parameter were observed in cells incubated with glyphosate metabolite—methylphosphonic acid at 5 mM (F_6;77_ = 8.14; P < 0.001).

After 24 h incubation, it was observed that PMIDA at 10 mM and bis-(phosphonomethyl)amine at 7.5 mM and 10 mM caused statistically significant changes in FSC-A parameter (F_5;48_ = 46.94; P < 0.001; F_5;48_ = 21.46; P < 0.001).

The analysis revealed that all compounds studied at different concentrations after 24 h incubation caused changes in SSC-A parameter. Changes in PBMCs granularity occurred from 0.5 mM of glyphosate (F_5;48_ = 66.54; P < 0.001); 5 mM of AMPA (F_5;48_ = 36.92; P < 0.001), *N*-methylglyphosate (F_5;48_ = 73.10; P < 0.001), methylphosphonic acid (F_5;48_ = 77.16; P < 0.001) and hydroxymethylphosphonic acid (F_5;48_ = 61.76; P < 0.001); 7.5 mM of bis-(phosphonomethyl)amine (F_5;48_ = 122.52; P < 0.001) and at 10 mM for PMIDA (F_5;48_ = 176.44; P < 0.001).

## Discussion

More than 900 million kilograms of herbicides, most of which is glyphosate enter the environment each year [16]. Glyphosate has been determined in the urine of humans at levels corresponding to a dietary daily intake of around 0.1–3.3 μg/kg bw/day [17]. High volumes of adjuvants (also called surfactants) are also used in herbicide preparations, and thus they (or their transformation products) are found in the environment [18] and food [19, 20].

Glyphosate’s re-registration has been expected in 2015. It has been recommended to increase the ADI level for glyphosate from 0.3 to 0.5 mg/kg bw/day [21]. The analysis of toxic effects of glyphosate, its metabolites and impurities seem to be very important to estimate health risk exerted by these compounds. As literature reports have revealed, the impurities in pesticide formulations, even at low concentrations may pose a greater risk than their parent compounds. It has been found that chlorinated dioxins and 2,4-dichlorophenol which are the impurities of the pesticides like 2,4-dichlorophenoxyacetic acid (2,4-D) and 2,4,5-trichlorophenoxyacetic acid (2,4,5-T) reveal stronger toxicity than their parent compounds [22]. Similarly, Sosnowska et al. [23] showed that dibromo-bromfenvinphos a contaminant of bromfenvinphos (BFV) induced stronger inhibition of aetylcholinesterase activity in human erythrocytes than BFV did.

In this work, the effect of glyphosate, its metabolites and impurities on human PBMCs viability, morphology and ATP level has been studied.

The study showed low cytotoxic potential of these compounds. IC_50_ values estimated for changes in cell viability and ATP level in PBMCs incubated with glyphosate, its metabolites and impurities for 24 h are shown in Table 1. The strongest changes were observed for PMIDA and bis-(phosphonomethyl)amine.

A decrease in ATP level promotes oxidative stress, which may lead to damage and dysfunction of various cells [24]. It was also proven that if ATP levels are markedly reduced in lymphocytes, these cells undergo necrosis [25]. That is why, it is probable that a decrease in viability in PBMCs viability exposed to high concentration of glyphosate, its metabolites and impurities was associated with ATP depletion.

Glyphosate and its derivatives did not induce any cytotoxic changes in the concentrations that may influence human organism environmentally or even occupationally exposed. Glyphosate was detected at a concentration of 73.6 ± 28.2 ng/ml (435 ± 166.7 μmol/l) in blood of humans exposed indirectly to this herbicide [26].

In the case of glyphosate intoxication, its content in blood was in the range from 0.6 to 150 mg/L (3.54 ± 887.21 μmol/l), whereas during moderate poisoning with this pesticide, it was detected in the concentrations from 690 (4.1 mmol/l) to 7480 mg/L (44.2 mmol/l) [27].

Our results clearly show that the compounds studied only at very high concentrations caused toxic effects in PBMCs. These concentrations correspond to those, which can enter human body only as a result of acute or subacute poisoning with glyphosate.

Benachur and Seralini [28] using MTT test analyzed the effect of glyphosate, AMPA and four glyphosate (Roundup) formulations on viability of 3 cells types, i.e. HUVEC cell line (cell line umbilical vein), human kidney cells 293, and cells derived from the placenta JEG3. For the compounds tested, the strongest toxicity was noted for Grands Travaux Roundup in which the glyphosate content reaches 400 g/l. The 24 hours incubation resulted in necrotic death of cells in all examined lines. The concentration, which initiated changes in the above cell lines was 0.002%, which corresponds to 47 μM of pure glyphosate. In turn, the glyphosate itself significantly affected cell viability at 100 μM. MTT test was also used by Young et al. [29]. They analyzed the impact of glyphosate and two its formulations on choriocarcinoma cell line. They estimated EC_50_ value for pure glyphosate and its commercial product, which was 16 mM and 8 mM, respectively.

Necrotic death is usually associated with alterations in cell morphology. Michałowicz et al. [30] observed necrotic changes in human PBMCs incubated with bisphenol A and its analogs, which were associated with alterations in size of this cell type. In our work, the analysis of the size and granularity of human PBMCs was performed by measuring forward (FSC-A) and side (SSC-A) light scatter characteristic using flow cytometry. The result showed that the strongest statistically significant changes were observed for PMIDA and bis-(phosphonomethyl)amine. The increase in the above mentioned parameters was 13% and 150% for PMIDA and 9% and 92% for bis-(phosphonomethyl)amine for FSC-A and SSC-A parameters, respectively.

The obtained results showed that glyphosate, its metabolites and impurities did not affect the above parameters in the concentration that may influence humans environmentally or occupationally exposed. The study revealed stronger toxicity of two metabolites such as PMIDA and bis-(phosphonomethyl)amine, which may be associated with complexation of metal divalent ions by these substances. Aiy et al. [31] showed that these compounds can bind divalent ions, including calcium. This phenomenon could explain a decrease in the ATP level observed in our study, which could have been linked with alterations in calcium ion level in this organellum.

Generally commercial preparations containing glyphosate cause stronger changes than glyphosate itself [32, 33]. These findings may be due to significant toxicity of surfactants present in the pesticide preparations [34]. The research conducted by Martinez et al. [33] on PBMCs have shown that cytotoxic effects caused by Roundup was stronger than induced by glyphosate. IC_50_ values determined for glyphosate formulation and pure glyphosate were estimated to be 1.64 mg/L and 56.4 mg/L, respectively.

Summing up, it has been proven that glyphosate in the concentrations examined, did not induce significant cytotoxic effect on PBMCs. Some of its impurities such as PMIDA and bis-(phosphonomethyl)amine exhibited stronger cytoxicity than their parent compound; nevertheless, they comprise a small contamination of the product and thus should not (in contrast to surfactants) increase glyphosate toxicity.

## Abbreviations

AMPA: aminomethylphosphonic acid;
PBMCs: peripheral blood mononuclear cells
PMIDA: *N-*(phosphonomethyl)iminodiacetic acid
